# The Influence of Drug Properties and Ontogeny of Transporters on Pediatric Renal Clearance through Glomerular Filtration and Active Secretion: a Simulation-Based Study

**DOI:** 10.1208/s12248-020-00468-7

**Published:** 2020-06-21

**Authors:** Sînziana Cristea, Elke Henriëtte Josephina Krekels, Amin Rostami-Hodjegan, Karel Allegaert, Catherijne Annette Jantine Knibbe

**Affiliations:** 1 Division of Systems Biomedicine and Pharmacology, Leiden Academic Center for Drug Research, Leiden University, Leiden, The Netherlands; 2 Simcyp Limited, Sheffield, UK; 3 Centre for Applied Pharmacokinetic Research (CAPKR), University of Manchester, Manchester, UK; 4 Clinical Pharmacy, Erasmus Medical Center, Rotterdam, The Netherlands; 5 Department of Development and Regeneration, KU Leuven, Leuven, Belgium; 6 Department of Pharmaceutical and Pharmacological Sciences, KU Leuven, Leuven, Belgium; 7 Department of Clinical Pharmacy, St. Antonius Hospital, Nieuwegein, The Netherlands

**Keywords:** active tubular secretion, glomerular filtration, ontogeny

## Abstract

Glomerular filtration (GF) and active tubular secretion (ATS) contribute to renal drug elimination, with the latter remaining understudied across the pediatric age range. Therefore, we systematically analyzed the influence of transporter ontogeny on the relative contribution of GF and ATS to renal clearance CL_R_ for drugs with different properties in children. A physiology-based model for CL_R_ in adults was extrapolated to the pediatric population by including maturation functions for the system-specific parameters. This model was used to predict GF and ATS for hypothetical drugs with a range of drug-specific properties, including transporter-mediated intrinsic clearance (CL_int,T_) values, that are substrates for renal secretion transporters with different ontogeny patterns. To assess the impact of transporter ontogeny on ATS and total CL_R_, a percentage prediction difference (%PD) was calculated between the predicted CL_R_ in the presence and absence of transporter ontogeny. The contribution of ATS to CL_R_ ranges between 41 and 90% in children depending on fraction unbound and CL_int,T_ values. If ontogeny of renal transporters is < 0.2 of adult values, CL_R_ predictions are unacceptable (%PD > 50%) for the majority of drugs regardless of the pediatric age. Ignoring ontogeny patterns of secretion transporters increasing with age in children younger than 2 years results in CL_R_ predictions that are not systematically acceptable for all hypothetical drugs (%PD > 50% for some drugs). This analysis identified for what drug-specific properties and at what ages the contribution of ATS on total pediatric CL_R_ cannot be ignored. Drugs with these properties may be sensitive *in vivo* probes to investigate transporter ontogeny.

**Electronic supplementary material:**

The online version of this article (10.1208/s12248-020-00468-7) contains supplementary material, which is available to authorized users.

## INTRODUCTION

Between 21 and 31% of marketed drugs are primarily renally cleared [1]. Processes underlying renal clearance (CL_R_) include glomerular filtration (GF), active tubular secretion (ATS), reabsorption, and renal metabolism. Maturation of GF has been extensively studied and quantified in children. However, less is known about the impact of maturation in the other process on CL_R_, partly due to the lack of specific biomarkers to distinguish between the activity of different transporters and to the overlap in specificity of transporters for different substrates Together with GF, ATS is one of the major contributing pathways for CL_R_; ontogeny of ATS is therefore the focus of the current analysis.

ATS involves different transporter systems located on the basolateral and apical sides of the proximal tubule cells of the kidney. These systems enable the efflux of drugs from the blood into the tubule where pre-urine is formed [2]. The expression of renal transporters was found to change in children [3]. However, these findings are based on a limited number of postmortem kidney samples collected throughout the pediatric age range [3]. Furthermore, there is limited information about the relationship between transporter-specific protein expression and transporter activity [4] or whether this remains constant with age. Finally, the extent to which transporter activity impacts ATS and subsequently total CL_R_ has not been quantified yet for the pediatric population.

Physiology-based pharmacokinetic (PBPK) models [5] integrate prior knowledge on drug and system properties. This configuration can be leveraged to perform extrapolations to unstudied scenarios. For example, PBPK models can be back-extrapolated to the pediatric population by taking into account the developmental changes in system parameters and be further used to make predictions in this special population for drugs that have not been studied in children yet. Previously, our group has used PBPK approaches in an innovative manner to systematically assess in which situations empirical scaling methods (i.e., allometric scaling, linear scaling) could be used to accurately scale plasma clearance of drugs that were eliminated by hepatic metabolism or GF for a broad range of hypothetical drugs [6, 7]. However, due to limited information on the ontogeny of renal transporters, the accuracy of clearance scaling for drugs eliminated through ATS could not be addressed.

Using a similar PBPK-based modeling approach as the one described above, we performed a systematic analysis to investigate the impact of the ontogeny of renal secretion transporters in relation with maturation of other physiological processes on the relative contribution of GF and ATS to CL_R_ as well as on the total CL_R_. This assessment was performed throughout the pediatric age range for a large number of hypothetical drugs with different properties covering a realistic parameter space. Moreover, to assess the impact of renal transporter ontogeny on CL_R_ throughout the pediatric population, we compared CL_R_ predictions obtained with and without including ontogeny patterns for renal transporters.

## METHODS

### Expansion of a PBPK Framework to Predict CL_R_ in Children

For this simulation study, a PBPK-based framework was developed analogue to the one published by Calvier *et al.* for plasma clearance by liver metabolism, and GF [6]. R v3.5.0 under R studio 1.1.38 was used to build the framework and to perform the systematic simulations.

An existing PBPK model for predicting CL_R_ in adults [5] was extrapolated to the pediatric population by incorporating published maturation functions for the system-specific parameters in the model. The model assumes a serial arrangement of the two major contributing pathways, GF and ATS (Eq. 1):

1 $$ \mathrm{C}{\mathrm{L}}_{\mathrm{R}}={\mathrm{CL}}_{\mathrm{GF}}+{\mathrm{CL}}_{\mathrm{ATS}}=\kern0.5em {f}_u\times \mathrm{GFR}+\kern0.5em \frac{\left({Q}_R-\mathrm{GFR}\right)\times {f}_u\times C{L}_{\operatorname{int},\sec }\ }{Q_R+{f}_u\times \frac{C{\mathrm{L}}_{\operatorname{int},\sec }}{\mathrm{BP}}} $$

where *CL*_*GF*_ and *CL*_*ATS*_ represent the clearance by GF and ATS, respectively, and *f*_*u*_ is the fraction unbound; *GFR* is the glomerular filtration rate; *Q*_*R*_ is renal blood flow; *BP* is the blood to plasma ratio of the drug; and *CL*_*int,sec*_ is the intrinsic secretion clearance of the active transporters. This model assumes that only the unbound drug in plasma is available for elimination, whereas drugs bound to plasma proteins or accumulated in erythrocytes are considered unavailable for elimination.

Maturation functions from literature were included for plasma concentrations of human serum albumin (HSA) and α-acid glycoprotein (AGP) [8], GFR [9], Q_R_ [10], hematocrit [10], kidney weight [10], and relative ontogeny for transporter-mediated intrinsic clearance (ont_T_). The functions for ont_T_ described either hypothetical values or published functions for individual [3] or aggregated [11, 12] transporter systems.

The concentrations of the two plasma proteins impact the f_u_ of the drug in plasma and the hematocrit levels impact BP. CL_int,sec_ is obtained as the product of transporter-mediated intrinsic clearance (CL_int,T_), ont_T_, the number of proximal tubule cells per gram kidney (PTCPGK), and kidney weight (KW), as shown in Eq. (2):

2 $$ \mathrm{C}{\mathrm{L}}_{\operatorname{int},\sec }=\mathrm{C}{\mathrm{L}}_{\operatorname{int},\mathrm{T}}\times \mathrm{on}{\mathrm{t}}_{\mathrm{T}}\times \mathrm{PTCPGK}\times \mathrm{KW} $$

**Fig. 1:**
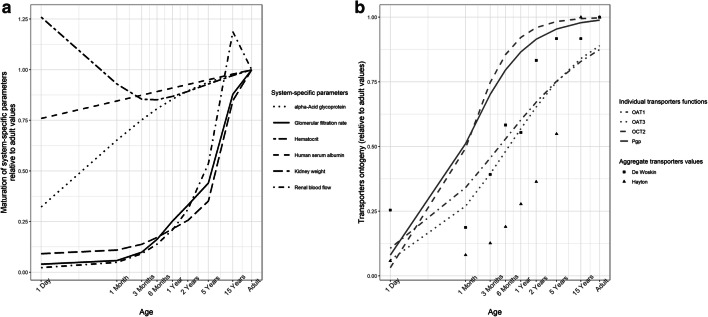
Published functions illustrating **a** the maturation of system-specific parameters and **b** age-dependent ontogeny functions (ontT) for individual or aggregated transporter systems used with the transporter-mediated intrinsic clearance (CLint,T) to obtain intrinsic secretion clearance (CLint,sec). These functions were used to extend the PBPK model to the pediatric population according to the functions in Table 1

CL_int,T_ is the resultant of expression and activity of renal secretion transporters. While maturation functions for KW and ont_T_ were included in the pediatric PBPK model for CL_*R*_, the number of proximal tubule cells per gram kidney was assumed to have the same value in children as in adults (60 × 10 [6] cells), as no information was available about its development. KW (g) was calculated across the pediatric age by multiplying the kidney volume (L) with a kidney density of 1050 g/L as obtained from Simcyp v18. All maturation functions and parameter values on which the PBPK model for CL_R_ is dependent can be found in Table 1. These maturation functions are depicted in Fig. 1a.

**Table 1:** Maturation functions used in Eqs. (1) and (2) for the extrapolation of system-specific and combined system-specific and drug-specific model parameters in the physiology-based pharmacokinetic (PBPK) model for renal clearance from typical adults to typical pediatric individuals

System-specific parameters for Eqs. (1) and (2) (abbreviation) (units)	Maturation functions included in the pediatric PBPK model for CL_R_
Glomerular filtration rate (GFR) (mL/min)	$\mathrm{GFR}=112\times {\left(\frac{\mathrm{WT}}{70}\right)}^{0.63}\times \left(\frac{\mathrm{PM}{\mathrm{A}}^{3.3}}{\mathrm{PM}{\mathrm{A}}^{3.3}+{55.4}^{3.3}}\right)$
Fraction unbound (f_u_) (-)	[HSA ]_ped/adult_ = 1.1287 × ln(AGE) + 33.746 ${\left[\mathrm{AGP}\right]}_{\mathrm{ped}/\mathrm{adult}}=\frac{0.887\times {\mathrm{AGE}}^{0.38}}{8.89^{0.38}+{\mathrm{AGE}}^{0.38}}$ $\to {\mathrm{f}}_{\mathrm{u},\mathrm{ped}}=\frac{1}{1+\frac{\left(1-{\mathrm{f}}_{\mathrm{u},\mathrm{adult}}\right)\times {\left[\mathrm{P}\right]}_{\mathrm{ped}}}{{\left[\mathrm{P}\right]}_{\mathrm{adult}}\times {\mathrm{f}}_{\mathrm{u},\mathrm{adult}}}\ }$
Renal blood flow (Q_R_) (mL/min)	CO = BSA × (110 + 184 × e^−0.0378 × AGE^ − e^−0.24477 × AGE^) $\mathrm{fr}=\frac{\mathrm{f}{\mathrm{r}}_{\mathrm{males}}+\mathrm{f}{\mathrm{r}}_{\mathrm{f}\mathrm{emales}}}{2}$ $\mathrm{f}{\mathrm{r}}_{\mathrm{males}}=4.53+\left(14.63\times \frac{\mathrm{AGE}}{0.1888+\mathrm{AGE}}\right)$ $\mathrm{f}{\mathrm{r}}_{\mathrm{females}}=4.53+\left(13\times \frac{{\mathrm{AGE}}^{1.15}}{0.188^{1.15}+{\mathrm{AGE}}^{1.15}}\right)$ →Q_R_ = CO × fr
Intrinsic secretion CL (CL_int,sec_) (mL/min)	PTCPGK = 60 (adult value) KW = 1050 × (4.214 × WT^0.823^ + 4.456 × WT^0.795^)/1000 **→**CL_int, sec_ = ont_T_ × CL_int, T_ × PTCPGK × KW
Blood to plasma ratio (BP) (-)	$\mathrm{hemat}=\frac{\mathrm{hema}{\mathrm{t}}_{\mathrm{male}}+\mathrm{hema}{\mathrm{t}}_{\mathrm{female}}}{2}$ $\mathrm{hema}{\mathrm{t}}_{\mathrm{male}}=53-\left(\left(43\times \frac{{\mathrm{AGE}}^{1.12}}{0.05^{1.12}+{\mathrm{AGE}}^{1.12}}\right)\times \left(1+\left(-0.93\times \frac{{\mathrm{AGE}}^{0.25}}{0.10^{0.25}+{\mathrm{AGE}}^{0.25}}\right)\right)\right)$ $\mathrm{hema}{\mathrm{t}}_{\mathrm{female}}=53-\left(\left(37.4\times \frac{{\mathrm{AGE}}^{1.12}}{0.05^{1.12}+{\mathrm{AGE}}^{1.12}}\right)\times \left(1+\left(-0.80\times \frac{{\mathrm{AGE}}^{0.25}}{0.10^{0.25}+{\mathrm{AGE}}^{0.25}}\right)\right)\right)$ →BP = 1 + hemat × (f_u_ × k_p_ − 1)
Published ontogeny functions for renal transporters (ont_T_) (-)	$\mathrm{on}{\mathrm{t}}_{\mathrm{P}-\mathrm{gp}}=\frac{\mathrm{P}\mathrm{N}{\mathrm{A}}^{0.73}}{\mathrm{P}\mathrm{N}{\mathrm{A}}^{0.73}+{4.02}^{0.73}}$ $\mathrm{on}{\mathrm{t}}_{\mathrm{OA}{\mathrm{T}}_1}=\frac{\mathrm{PN}{\mathrm{A}}^{0.43}}{\mathrm{PN}{\mathrm{A}}^{0.43}+{19.71}^{0.43}}$ $\mathrm{on}{\mathrm{t}}_{\mathrm{OA}{\mathrm{T}}_3}=\frac{\mathrm{PN}{\mathrm{A}}^{0.51}}{\mathrm{PN}{\mathrm{A}}^{0.51}+{30.70}^{0.51}}$ $\mathrm{on}{\mathrm{t}}_{\mathrm{OC}{\mathrm{T}}_2}=\frac{\mathrm{PN}{\mathrm{A}}^1}{\mathrm{PN}{\mathrm{A}}^1+{4.38}^{0.51}}$ $\ast \mathrm{o}{\mathrm{nt}}_{\mathrm{AT}{\mathrm{S}}_{\mathrm{Hayton}}}=\frac{\left(1.08\times \mathrm{weigh}{\mathrm{t}}^{1.04}\times {\mathrm{e}}^{-0.185\times \mathrm{age}}+1.83\times \mathrm{weigh}{\mathrm{t}}^{1.04}\times \left(1-{\mathrm{e}}^{-0.185\times \mathrm{age}}\right)\right)}{\mathrm{o}{\mathrm{nt}}_{\mathrm{AT}{\mathrm{S}}_{\mathrm{Hayton}}}\left(\mathrm{adult}\right)}$ $\ast \mathrm{on}{\mathrm{t}}_{\mathrm{AT}{\mathrm{S}}_{\mathrm{DeWoskin}}}=\frac{20.3}{79.8},\frac{14.9}{79.8},\frac{31.3}{79.8},\frac{46.5}{79.8},\frac{44.2}{79.8},\frac{66.5}{79.8},\frac{73.15}{79.8},\frac{73.15}{79.8},\frac{79.8}{79.8}$, at 1 day, 1 month, 3 months, 6 months, 1 year, 2 years, 5 years, 15 years, and adult, respectively

*WT* bodyweight (kg); *PMA* postmenstrual age (weeks); *HSA* human serum albumin (g/L); *AGP* α-acid glycoprotein (g/L); *P* plasma-binding protein (e.g. HSA or AGP (g/L); *CO* cardiac output (mL/min); *hemat* hematocrit; *fr* fraction of cardiac output directed to renal artery; *BSA* body surface area (m2); *AGE* age in (days) for the maturation of (P) and in (years) for the fraction of cardiac output and hematocrit levels; *PTCPGK* proximal tubule cells per gram kidney (× 106 cells); *KW* kidney weight (g); *ontT* transporters ontogeny relative to adult levels (−); *CLint,T* transporter-mediated active clearance (mL/min); *kp* blood-to-plasma partitioning coefficient of a drug; *PNA* postnatal age (weeks*)*

*Hayton *et al.* developed a continuous function using age in years and weight in kg, based on the data published by Rubin *et al.* [17]. The function covers the pediatric age range up to 12 years and values obtained at 12 years were considered mature and assigned to the typical 15-year-old and adult (ont_ATS-Hayton_(adult))

*DeWoskin *et al.* collected literature data on tubular secretion rates and categorized it in different age groups, from neonates up to adults. For children older than 1 year and younger than 18 years, the average between the values published for children and adults was interpolated

Ont_T_ is included in Eq. (2) as a fraction relative to the adult CL_int,T_. In this way, pediatric CL_int,T_ [1] remained fixed at the adult CL_int,T_ levels (ont_T_ = 1, meaning ontogeny is absent), [2] was a constant fraction of the adult CL_int,T_ throughout the entire pediatric age range, or (3) increased with age as flexible fraction of adult CL_int,T_ according to published ontogeny functions [3]. For the relative ontogeny fractions that remained constant throughout the pediatric age, the following values were used: 0.05, 0.2, 0.5, and 0.7. Ontogeny functions that increased with age were taken from literature, including 4 functions for individual transporters [3] (i.e., OAT1, OAT3, OCT2, and Pgp) and 2 functions for aggregated transporter systems [11, 12]. All the relative ontogeny functions for CL_int,T_ that increased with age, and the details about their implementation in the model are presented in Table 1. In addition, the published ontogeny functions that characterize relative ontogeny for individual (i.e., OAT1, OAT3, OCT2, and Pgp) and aggregated (i.e., Hayton *et al.*, DeWoskin *et al.*) transporters throughout the pediatric population relative to adult values are visualized in Fig. 1b.

The pediatric PBPK-based model was used to predict CL_R_ in typical virtual individuals. For this, patients with the following ages were selected: 1 day, 1, 3, and 6 months, and 1, 2, 5, and 15 years for pediatric individuals and 35 years for the adult. The demographics for the typical pediatric individuals required to obtain the maturation functions in the PBPK-based model were derived from the NHANES database [13], and the ones for the typical adult were derived from the ICRP annals [14]. The demographic characteristics corresponding to these ages are given in Table 2.

**Table 2:** Demographics of the typical virtual pediatric individuals [13] and adult [14] included in this analysis

Age	Height (cm)	Weight (kg)	Hematocrit (%)	Body surface area (m^2^)
1 day	49.75	3.5	56	0.22
1 month	54.25	4.3	44	0.25
3 months	60	5.75	35.5	0.31
6 months	66	7.55	36	0.37
1 year	74.75	9.9	36	0.46
2 years	86	12.35	36.5	0.54
5 years	108.25	18.25	37	0.73
15 years	166	54.25	42	1.59
Adult	169.5	66.5	44	1.76

For a systematic investigation of the drug-specific parameter space, hypothetical drugs with different properties were generated, and their CL_R_ was predicted with the PBPK model for CL_R_ for all typical individuals. The hypothetical drugs were defined by four drug-specific properties for which ranges of realistic values were used as follows:

1. The drugs were assumed to bind exclusively to either HSA or AGP.
2. f_u,adult_ values of 0.05, 0.15, 0.25, 0.35, 0.45, 0.55, 0.65, 0.75, 0.85, 0.95, and 1 were used for drugs binding to either HSA or AGP.
3. BP was obtained from hematocrit levels and *Kp* values in adults of 0, 1, 2, 3, and 4 (Table 1) [15].
4. For CL_int,T_ 39 representative values were sampled within the range of 2 and 500 mL/min/mg protein. The selected range was based on CL_int,T_ values obtained from published CL_R_ values in adults following retrograde calculation for 53 drugs that are renally excreted by ATS. The obtained CL_int,T_ represents the affinity of the drug for different transporters together with the abundances of transporters. Details about the retrograde calculation of CL_int,T_ are shown in the Supplement section S1: Retrograde-calculation of CL_int,T_ from adult CL_R_ values, and the obtained CL_int,T_ values for these drugs in adults are displayed in Fig. S1.

Generating all possible combinations between the values given to the four drug properties yielded 3800 hypothetical drugs that were included in the current systematic analysis.

### Contribution of GF and ATS to Pediatric CL_R_ for Drugs with Different Properties

The PBPK framework was used to simulate CL_R_ for the 3800 hypothetical drugs for each typical virtual individual. Simulations with a relative ontogeny fixed at adult levels (ont_T_ = 1) were used to assess the impact of drug-specific properties on CL_R_ in the absence of transporter ontogeny. For each drug, the relative contribution of GFR and ATS to CL_R_ is determined according to Eqs. (3) and (4), respectively.

3 $$ \mathrm{GF}{\mathrm{R}}_{\mathrm{contribution}}\%=\frac{\mathrm{C}{\mathrm{L}}_{\mathrm{GFR}}}{\mathrm{C}{\mathrm{L}}_{\mathrm{R}}}\times 100 $$

4 $$ \mathrm{AT}{\mathrm{S}}_{\mathrm{contribution}}\%=\frac{\mathrm{C}{\mathrm{L}}_{\mathrm{ATS}}}{\mathrm{C}{\mathrm{L}}_{\mathrm{R}}}\times 100 $$

### Influence of Renal Transporters Ontogeny on Pediatric CL_R_

To assess the influence of ontogeny of kidney transporters on pediatric CL_R_, we implemented transporter ontogeny fractions relative to adult values in the pediatric PBPK model for CL_R_ (Eqs. (1) and (2)) such that ontogeny of CL_int,T_: [1] remained fixed at adult levels, [2] was a constant fraction of adult values throughout the pediatric age range, or (3) increased with age as a flexible fraction of adult values. The use of these implementations to describe the ontogeny of transporters enabled us to explore different values and patterns for transporter ontogeny to ultimately quantify the impact of these changes on ATS and CL_R_ throughout the pediatric age range. To quantify the influence of transporter ontogeny on pediatric CL_R_ predictions, a percentage prediction difference (%PD) was calculated between CL_R_ predictions without ontogeny (CL_R adult,ont,T_) (i.e., ont_T_ = 1) and CL_R_ predictions with transporter ontogeny that either remained constant or increased with age (CL_R pediatric, ont,T_) according to Eq. (5):

5 $$ \%\mathrm{PD}=\frac{\mathrm{C}{\mathrm{L}}_{{\mathrm{R}}_{\mathrm{adult},\mathrm{on}{\mathrm{t}}_{\mathrm{T}}}}-\mathrm{C}{\mathrm{L}}_{{\mathrm{R}}_{\mathrm{pediatric},\mathrm{on}{\mathrm{t}}_{\mathrm{T}}}}}{\mathrm{C}{{\mathrm{L}}_{\mathrm{R}}}_{\mathrm{pediatric},\mathrm{on}{\mathrm{t}}_{\mathrm{T}}}} \times 100 $$

The %PD obtained upon ignoring the ontogeny of kidney transporters was classified as leading to acceptable CL_R_ predictions for %PD below 30%, reasonably acceptable CL_R_ predictions for %PD between 30 and 50%, and unacceptable CL_R_ predictions for %PD above 50%. As published transporter ontogeny patterns only increase with age (i.e., ont_T_ is always between 0 and 1) until they reach adult CL_int,T_ levels (i.e., ont_T_ = 1), the %PD will always be positive.

In addition, %PD was used to assess the systematic accuracy of CL_R_ predictions obtained while ignoring transporter ontogeny. CL_R_ at a certain age would have systematically acceptable predictions for a transporter pathway when the maximum %PD value for all 3800 hypothetical drugs at that pediatric age was below 30%. In this case, ontogeny of transporters was expected to have a limited role in predicting CL_R_ for any drug at that age. When CL_R_ predictions obtained in the absence of transporter ontogeny were reasonably acceptable or unacceptable for one or more hypothetical drugs, CL_R_ predictions were no longer considered systematically acceptable. In this case, CL_R_ predictions might still be acceptable for some of the hypothetical drugs; however, it cannot be known a *priori* whether CL_R_ predictions are acceptable or not for individual drugs, without taking drug properties into account. As such, systematically acceptable scenarios were a means to identify the pediatric ages for which the ontogeny of individual or aggregated transporters cannot be ignored, as it could lead to biased CL_R_ predictions.

## RESULTS

### Contribution of GF and ATS to Pediatric CL_R_ for Drugs with Different Properties

**Fig. 2:**
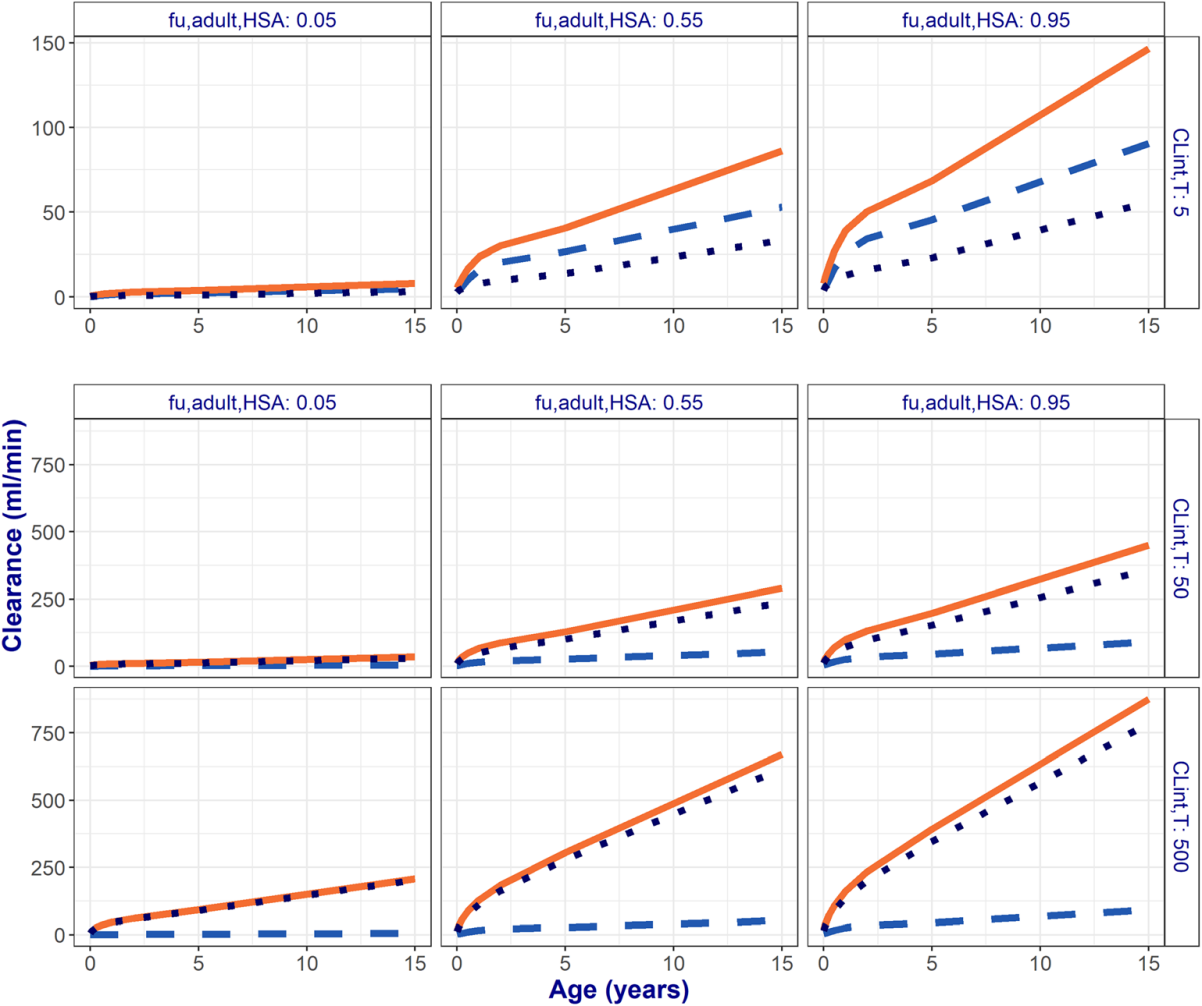
Developmental changes in total renal clearance (CL_R_, solid orange lines) and the contribution of glomerular filtration (GF, light blue dashed lines) and active tubular secretion (dark blue dotted lines) *vs.* age for 9 representative hypothetical drugs. These drugs bind to albumin (HSA) and have low, medium, or high unbound fractions in adults (f_u,adult_, horizontal panels) that change with age, dependent on the HSA plasma concentrations. Transporter-mediated intrinsic clearance values (CL_int,T_) were assumed to remain constant with age at the indicated values (vertical panels).Note the different scales on the *y*-axes for the graphs in the top row (range 0–150 mL/min) compared with the middle and bottom row (range 0–750 mL/min)

The contributions of GF and ATS to CL_R_ over age are shown in Fig. 2 for a selection of 9 hypothetical drugs with varying CL_int,T_ and f_u,adult_ values. These drugs represent the mean and the extremes of the assessed ranges for these parameter values. Here ont_T_ was fixed at 1, meaning that results show the influence of maturation of system-specific parameters other than transporter ontogeny on CL_R_. Very similar results were obtained for drugs binding to AGP (Fig. S2).

Figure 2 and Fig. S2 show that GF and ATS increase nonlinearly throughout the pediatric age range with the steepest increase in the first year of life and continue to increase moderately up to the age of 15 years. Clearance by GF is strictly dependent on the maturation of GFR and on the concentrations of drug-binding plasma proteins, which impact f_u_. Clearance by ATS changes with age, and it depends on the maturation of Q_R_, KW, concentrations of drug-binding plasma proteins, and hematocrit levels, the latter of which impact BP (Table 1).

The relative contribution of GF and ATS to CL_R_ is strongly impacted by CL_int,T_. For drugs mainly cleared by GF (e.g., CL_int,T_ = 5 μL/min/mg protein), the relative contribution of ATS to CL_R_ is on average 41%, and it decreases with age from 52% in neonates to 35% between ages 2 and 15 years. As CL_int,T_ increases, ATS becomes the main pathway for CL_R_. A 10-fold increase in CL_int,T_ from 5 to 50 μL/min/mg protein increases the relative contribution of ATS, on average, from 41 to 80%. When CL_int,T_ is further increased up to 500 μL/min/mg protein, ATS relative contribution increases up to 90%.

Changes in CL_R_ are dependent on age-related changes in system-specific parameters as well as on differences in drug-specific parameters. Drugs mainly cleared by GF (e.g., CL_int,T_ = 5 μL/min/mg protein) show, on average, a 15-fold increase in CL_R_ (from 3 to 46 mL/min) with f_u,adult_ increasing from 0.05 to 0.95. For drugs mainly cleared by ATS with a CL_int,T_ of 50 μL/min/mg protein, the same increase in f_u,adult_ yields, on average, a 12-fold increase in CL_R_ (from 11 to 130 mL/min). For drugs that are mainly cleared by ATS and are largely unbound from plasma proteins (f_u,adult_ = 0.95), a 10-fold increase in CL_int,T_ (from 5 to 50 μL/min/mg protein) yields, on average, a 2.8-fold increase in CL_R_ (from 46 to 130 mL/min). For drugs with very high CL_int,T_ values, the same fold difference in CL_int,T_ (from 50 to 500 μL/min/mg protein) yields, on average, a lower increase in CL_R_ of only 1.8-fold (from 130 to 238 mL/min).

Changes in Kp (and implicitly in BP) may only become moderately relevant for drugs with very large CL_int,T_ values and medium to high f_u,adult_ values. When Kp increases from 1 to 4, CL_R_ increased, on average, only by 1.15-fold for drugs with CL_int,T_ = 50 μL/min/mg protein and f_u,adult_ = 0.55 and reached a maximum increase of 1.25-fold for drugs with CL_int,T_ = 500 μL/min/mg protein and f_u,adult_ = 0.95.

### Influence of Renal Transporters Ontogeny on CL_R_

The role of transporter ontogeny on CL_R_ was quantified by calculating the %PD between CL_R_ predictions with the transporter relative ontogeny fixed at adult levels (CL_R adult,ontT_, ont_T_ = 1) and CL_R_ predictions with relative transporter ontogeny that either remains at a constant fraction of adult values or increases over age for individual transporters, as published for OAT1, OAT3, OCT2, Pgp [3], and aggregated transporters [11, 12] (CL_R pediatric,ontT_).

**Fig. 3:**
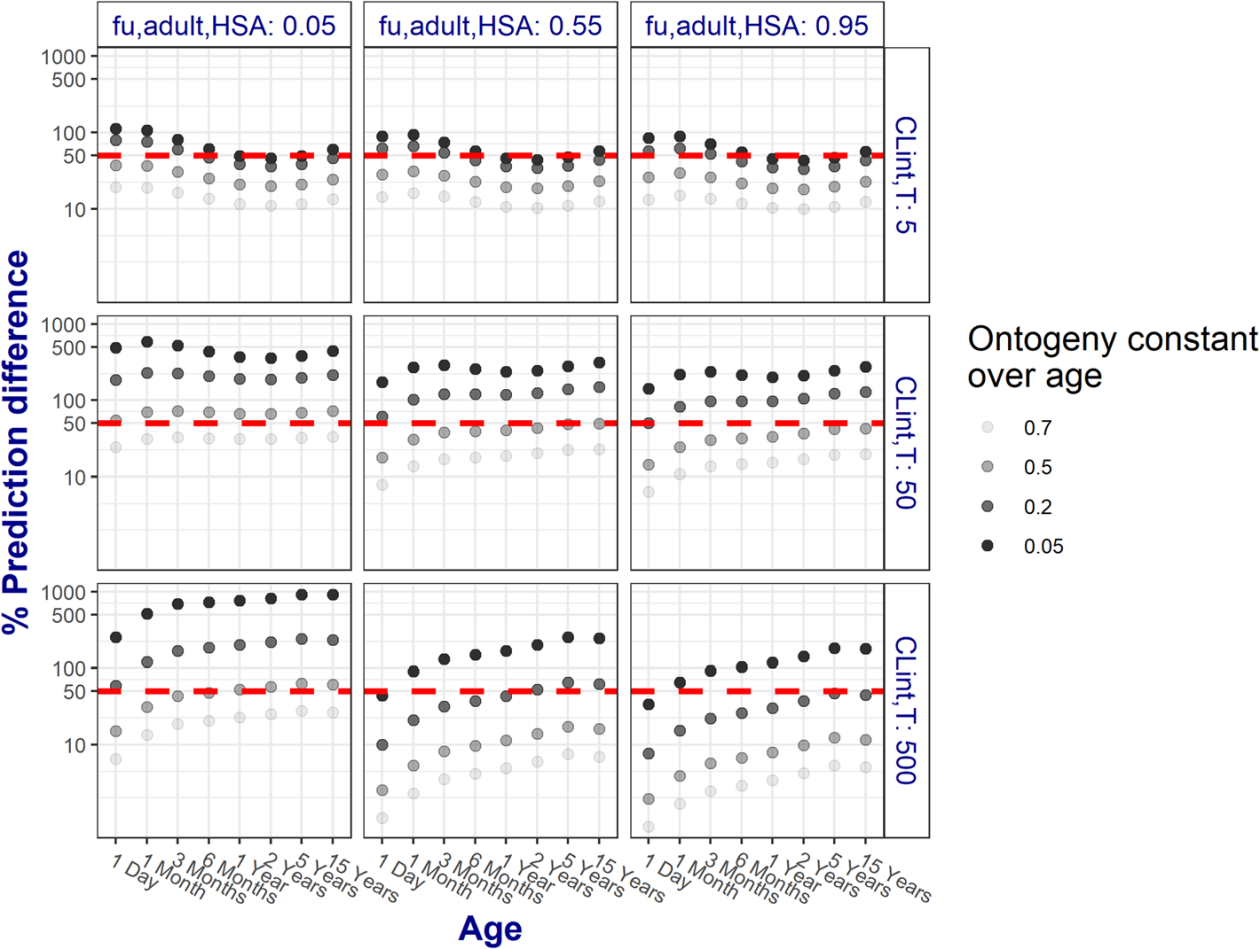
Percentage prediction difference (%PD) for 9 representative hypothetical drugs calculated between renal clearance (CL_R_) predictions obtained with the pediatric renal PBPK model that included or excluded hypothetical transporter ontogeny (ont_T_) values that remained constant over age. These hypothetical drugs bind to albumin (HSA) and have low, medium, or high unbound fractions in adults (f_u,adult_, horizontal panels) that change with age, dependent on the HSA plasma concentrations. Transporter-mediated intrinsic clearance values (CL_int,T_) were assumed to remain constant with age at the indicated values (vertical panels). The colors of the %PD increase with decreasing transporter ontogeny values (ont_T_). The dashed red line represents the threshold of reasonably acceptable CL_R_ prediction of 50%. Results are displayed on a log-log scale

Figure 3 shows the results for the same 9 hypothetical drugs as in Fig. 2, with four age-constant ontogeny fractions for the renal transporters (i.e., ont_T_ = 0.05, 0.2, 0.5, 0.7). Similar results are observed for drugs binding to AGP (Fig. S3). When transporters are underdeveloped (ont_T_ < 0.2), ontogeny of renal transporters cannot be ignored as it would lead to unacceptable CL_R_ predictions for all investigated hypothetical drugs regardless of age. The shapes of the %PD profiles for the 9 selected drugs differ from one another, depending on whether the primary elimination pathway contributing to CL_R_ is GF or ATS. This is related to the maturation of other system-specific parameters that are underlying GF and ATS.

For drugs that are mainly cleared by GF (CL_int,T_ = 5 μL/min/mg protein), in children younger than 6 months and relative transporter ontogeny lower than 0.2, ignoring ontogeny of kidney transporters would lead to unacceptable CL_R_ predictions (%PD = 53–113%). For children older than 6 months, with relative ontogeny higher than 0.05, reasonably acceptable CL_R_ predictions are obtained for all drugs mainly cleared by GF.

For drugs that are mainly cleared by ATS and have a low fraction unbound (CL_int,T_ ≥ 50 μL/min/mg protein with f_u,adult_ = 0.05) ignoring the ontogeny of transporters would lead to unacceptable CL_R_ predictions (%PD, 53–918%) for all pediatric individuals with a low transporter ontogeny (ont_T_ ≤ 0.5). For drugs with CL_int,T_ = 50 μL/min/mg protein and increasing f_u,adult_, reasonably acceptable CL_R_ predictions are obtained for all ages when relative transporter ontogeny is high (ont_T_ > 0.5). For these drugs, %PD can reach values between 50 and 316% when transporter ontogeny is low (ont_T_ ≤ 0.2). For drugs with a very large CL_int,T_ and high f_u,adult_ (CL_int,T_ = 500 μL/min/mg protein with f_u,adult_ = 0.95), the influence of transporter ontogeny on CL_R_ decreases, as indicated by the reasonably acceptable %PD values.

**Fig. 4:**
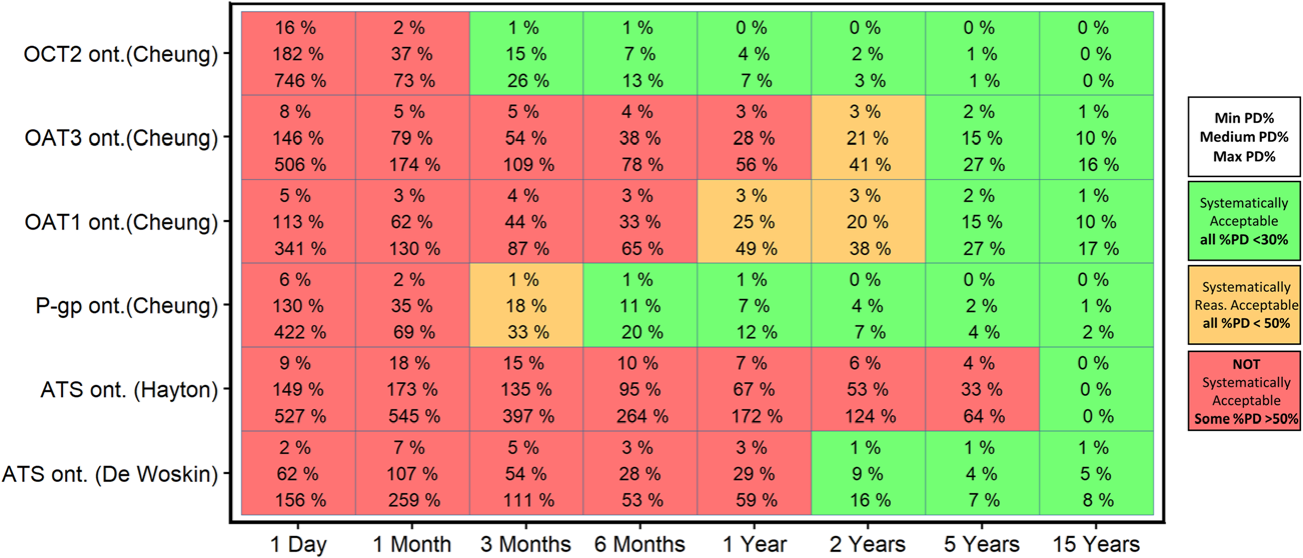
Percentage prediction difference (%PD) between CL_R_ predictions obtained with the pediatric PBPK model that does not include transporter ontogeny (ont_T_ = 1, reflecting adult values) and the model that includes age-specific pediatric ont_T_ values for each of the indicated transporter systems. In each box, the minimum (top), median (middle), and maximum (bottom) %PD is displayed to summarize the findings for all hypothetical drugs per typical pediatric individual at different ages. Systematically acceptable scenarios have %PD for all drugs < 30% (green box), reasonable acceptable scenarios have %PD for all drugs ≤ 50% (orange box), and the absence of systematic acceptance means that at least one drug has a %PD > 50% (red box)

The results shown in Fig. 4 complement the previous findings by illustrating the implications for CL_R_ predictions for drugs that are substrates for transporters for which ontogeny functions have been published. Figure 4 shows when CL_R_ predictions are systematically acceptable with or without transporter ontogeny functions (i.e., CL_R_ values obtained with ont_T_ values varying with age according to individual [3] or aggregated [11, 12]transporter functions for ontogeny and CL_R_ values obtained with ont_T_ fixed to the adult levels (ont_T_ = 1)). In both simulations, system-specific parameters and transporter ontogeny functions changed with age as shown in the Table 1 and Fig. 1*.*

Figure 4 displays the results as a heat map, where the numbers in each box represent the minimum, median, and maximum %PD values obtained for all 3800 hypothetical drugs that are substrates for the indicated individual transporter or aggregated transporters at every pediatric age. Systematically acceptable scenarios are achieved when CL_R_ predictions for all 3800 hypothetical drugs lead to %PD values below 30% in the absence of transporter ontogeny. This is indicated by the green boxes, while orange and red boxes indicate CL_R_ predictions that are reasonably acceptable (highest %PD between 30 and 50%) and unacceptable (highest %PD > 50%), respectively, for one or more drugs. Nonetheless, when CL_R_ predictions are not systematically acceptable, it does not imply that %PD values below 30% were not observed, rather it indicates that predictions for one or more drugs are biased at the indicated age. Hence, it cannot be predicted a *priori* whether the predictions without including ontogeny of transporters will be acceptable or not, without taking drug properties into account.

When the relative transporter ontogeny varied with age according to the functions of Cheung *et al.* (i.e., for OAT1, OAT3, OCT2, and P-gp) [3], ignoring ontogeny leads to CL_R_ predictions that were not systematically acceptable for all transporters in newborns of 1 month and younger. CL_R_ predictions of drugs that are substrates of OAT transporters are not systematically acceptable below the age of 1 year. For children of 2 years and older, ignoring the ontogeny of transporters leads to CL_R_ predictions that were reasonably acceptable or acceptable for all transporters―individual or aggregated―and all substrates, except when ontogeny follows the aggregated transporters ontogeny function as published by Hayton *et al.*.

## DISCUSSION

A PBPK-based framework was used to predict CL_R_ of hypothetical drugs with various properties that are substrates for renal secretion transporters throughout the pediatric age range. This approach provided insight on the contribution of GF and ATS to the total pediatric CL_R_. In addition, the impact of ignoring this transporter ontogeny in predicting CL_R_ in children was quantified.

The physiology-based model for CL_R_ used in the presented framework was developed based on a model published for adults [5] that was extended to the pediatric population by including maturation functions for the system-specific parameters as shown in Table 1 and illustrated in Fig. 1a. This model included two major contributing pathways to CL_R_: GF and ATS. Based on this model, we could quantify the impact of transporter ontogeny on pediatric drug clearance for all current and future small molecule drugs, based on drug-specific properties alone. We found that the contribution of these pathways to CL_R_ increases nonlinearly throughout the pediatric age range, with the steepest increase during the first year of life, even in the absence of transporter ontogeny. These changes in pediatric CL_R_ are determined by the influence of maturation in the system-specific parameters underlying GF and ATS as well as by drug-specific properties (Fig. 2). Both GF and ATS increase with increasing f_u_, while ATS also increases with increasing CL_int,T_ values.

**Fig. 5:**
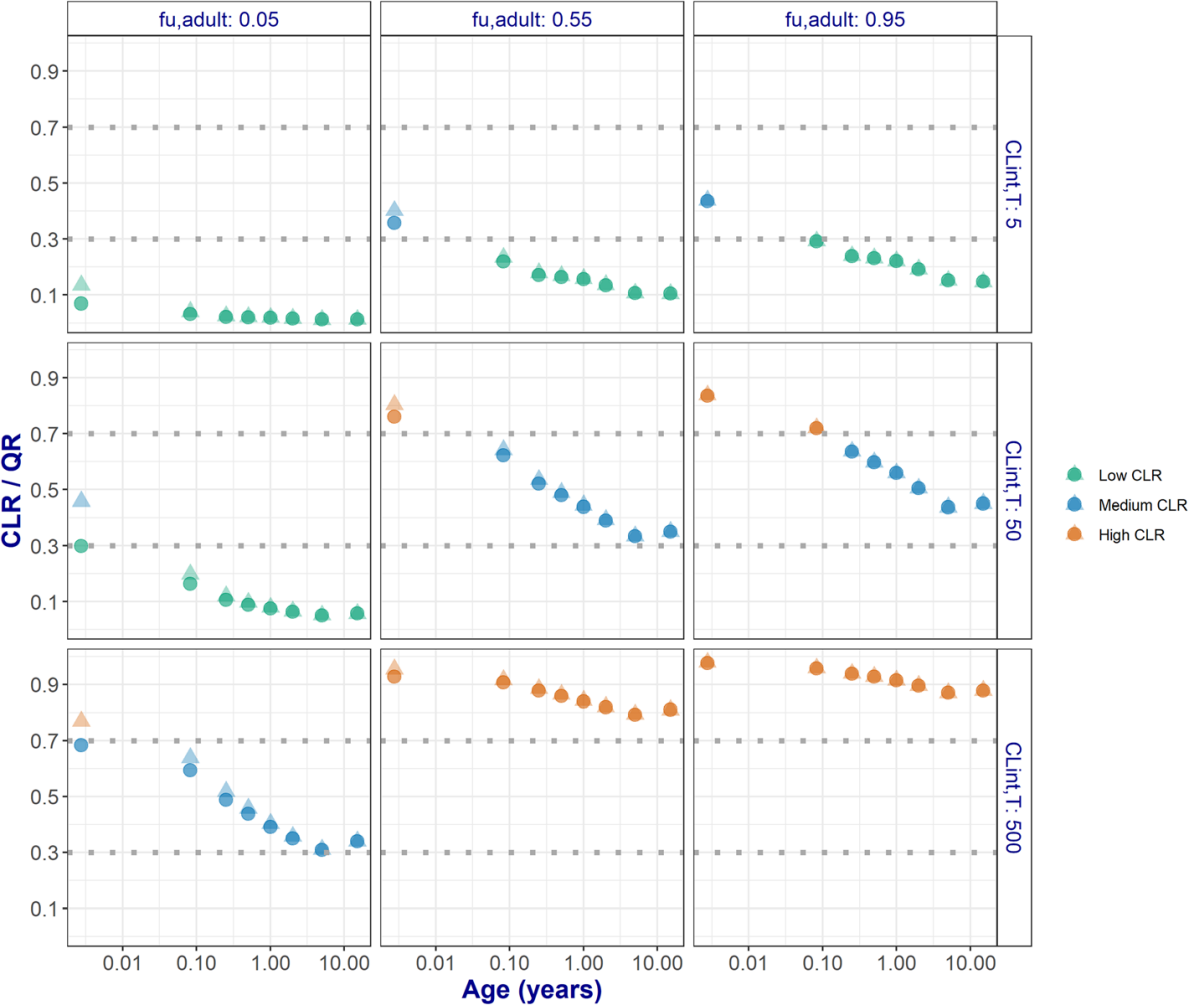
Ratio of total renal clearance (CLR) and renal blood flow (Q) for 9 representative hypothetical drugs. Results are presented for drugs binding to human serum albumin (HSA) (circles) or to α-acid glycoprotein (AGP) (faded triangles)

Drug f_u_ was found to have a major influence on CL_R_ through both investigated pathways but especially on CL_R_ through GF. CL_int,T_ has a major influence on CL_R_ only through ATS. Drugs with 10-fold different CL_int,T_ values and low binding to plasma proteins (f_u,adult_ = 0.95) yield different contributions of ATS to CL_R_. When ATS contribution to CL_R_ is limited only by the activity and the abundance of transporters (i.e., CL_int,T_ changes between 5 and 50 μL/min/mg protein), an increase of 1.9-fold in average ATS contribution was observed. As CL_int,T_ changes between 50 and 500 μL/min/mg protein, we observed a lower increase in average ATS contribution of only 1.1-fold [16]. This behavior could be explained by the fact that f_u_ and CL_int,sec_ are rate limiting factors for ATS when CL_int,sec_ x f_u_ is low relative to Q_R_ (i.e., permeability-limited process). Q_R_ becomes the rate limiting factor for ATS when CL_int,sec_ x f_u_ becomes highl in comparison to Q_R_ (i.e., perfusion-limited process).This also explains why the impact of ignoring transporter ontogeny decreases for drugs with very high CL_int,T_, as shown by the lower %PD values in Fig. 3. It is important to mention that the process limiting ATS may change with age, whether ATS is permeability-limited (CL_R_/Q_R_ < 0.3) or perfusion-limited (CL_R_/Q_R_ > 0.7) or a combination between the two processes, 0.3 < CL_R_/Q_R_ < 0.7, as shown in Fig. 5.

The present framework explored a broad parameter space for ontogeny of transporters. By keeping ontogeny of transporters constant with age, the potential impact of ignoring ontogeny on predicting CL_R_ was systematically explored (Fig. 3). This exploration highlights that an ontogeny below 0.2 of the adult value cannot be ignored for the majority of drugs regardless of the pediatric age. In this situation, the assumption that there are no differences in transporter ontogeny between children and adults would lead to unacceptable CL_R_ predictions. Data characterizing how ontogeny of individual kidney transporters changes across the pediatric age is scarce in literature. Cheung *et al.* [3] recently took the first steps in quantifying the ontogeny of protein abundance for individual renal transporters. According to this report, which is based on a limited sample size, BCRP, MATE1, MATE2-K, and GLUT2 have protein abundance levels similar to the adult levels throughout the studied pediatric age range [3], meaning that ont_T_ = 1 for children of all ages and that transporter ontogeny is not a factor of influence in predicting CL_R_ for substrates of these transporters. Including these ontogeny profiles in the current framework increased our understanding on the role of age-dependent ontogeny in predicting CL_R_ (Fig. 4). As reported by Cheung *et al.*, the ontogeny of OAT1 and OAT3 is slower than the ontogeny of OCT2 and P-gp. Ignoring OCT2 ontogeny yields systematically acceptable pediatric CL_R_ values for all its hypothetical substrates in children from 3 months and older. For P-gp substrates, the same holds true in children from 6 months and older. Ontogeny of OATs however cannot be ignored for children younger than 2 years as CL_R_ predictions are not systematically acceptable for substrates of this transporter. The CL_R_ predictions obtained with the aggregate transporter function published by DeWoskin *et al.* [11] are in line with the results for OATs. The aggregate function of Hayton *et al.* [12] suggests a much slower ontogeny leading to CL_R_ predictions that are not systematically acceptable in children up to and including 5 years. CL_R_ predictions with Hayton *et al.* [ 12] diverge from the predictions obtained with the other transporter ontogeny functions since it was the first function to quantify the ontogeny of ATS and has a different profile than all the other studied functions. Disregarding ontogeny of transporters leads to over-predictions of CL_R_ in young patients. If these predicted CL_R_ values were used as the basis for pediatric dose adjustments, these could lead to overexposure to drugs and, eventually, increase the risk of toxic events.

As our analysis identifies drugs for which CL_R_ is sensitive to transporter ontogeny, the proposed framework can also be used to find and select drugs with relevant properties to serve as *in vivo* probes for the quantification of the ontogeny of transporters underlying ATS. From the results of the current analysis, we could conclude that the best probe drugs should have a CL_int,T_ of 5–50 μL/min/mg protein and medium to high fraction unbound in adults (f_u,adults_ = 0.55–0.95). Drugs for which GF is the main elimination pathway or drugs with extremely high CL_int,T_ that cause renal blood flow to be limiting for elimination will have a limited use in characterizing ontogeny profiles. These guidelines could be the basis for future research aiming to derive ontogeny of individual renal transporters *in vivo*.

Our results rely on the validity of the PBPK approach, which is currently considered the “gold standard” for clearance predictions in the absence of clinical data. This approach gives an overview of the impact of system- and drug-specific parameters on CL_R_. The explored arrays of ontogeny fractions and of drug properties were realistic; however, unrealistic combinations of drug properties could have been generated. As with the previously published hepatic PBPK framework [6], this analysis does not include measures for the variability or uncertainty of the parameters that constitute the PBPK model, to highlight the impact of system- and drug-specific changes in the absence of variability and uncertainty. Our approach could be extended for investigations on the impact of variability and uncertainty by including variability terms on the system-specific parameters and performing stochastic simulations. Finally, PBPK modeling is ideally suitable to study the impact of specific physiological processes in a way that is not possible *in vivo*. In the *in vivo* situation, studies are limited to drugs that are currently available on the market and prescribed to children. However, generally these drugs are not eliminated in totality by one single pathway. Moreover, the accuracy of these observations is impacted by aspects related to study design, sampling, and analytical methods. Our current model-based analysis is not impacted by these limitations. The physiology-based model for CL_R_ used here only included GF and ATS, but not passive permeability, reabsorption, or renal metabolism. This enabled the study of GF and ATS in isolation and reduced the noise and complexity of the results. The influence of ontogeny on transporters working in tandem or of reabsorption and kidney metabolism together with their dependencies on physiological properties, like pH at the tubule side, ionization, enzyme abundance, affinity, and maturation, could be explored in a similar manner in subsequent analyses.

## CONCLUSION

A PBPK-based framework was used to determine the role of drug properties and ontogeny of transporters in predicting pediatric CL_R_. The contribution of GFR to CL_R_ is influenced by drug f_u_. The contribution of ATS to CL_R_ is predominantly influenced by changes in f_u_ and CL_int,T_ for drugs with low and medium CL_int,T_ as well as by changes in Q_R_ for drugs with high CL_int,T_. Transporters play a major role in predicting CL_R_. Discordance in the CL_R_ predictions when ignoring maturation in ATS shows when acceptable predictions of total pediatric CL_R_ from the adults if extrapolation solely relied on changes in GF with age are not possible. Ignoring transporter ontogeny, especially when it is below 0.2 of the adult values, leads to unacceptable CL_R_ predictions for the majority of drugs, regardless of age. Given known age-dependent patterns, transporter ontogeny cannot be ignored in children younger than 2 years. Drugs with properties that lead to high %PD when ignoring ATS ontogeny may serve as sensitive *in vivo* probes to further investigate transporter ontogeny.

## Electronic supplementary material

ESM 1 (DOC× 1062 kb)
