# Supplementary material for: The Influence of Drug Properties and Ontogeny of Transporters on Pediatric Renal Clearance through Glomerular Filtration and Active Secretion: a Simulation-Based Study
Source: AAPS J. 2020 Jun 21;22(4):87. doi: 10.1208/s12248-020-00468-7 (PMC7306484; doi:10.1208/s12248-020-00468-7)
Supplement: Supplementary file 1 — (DOC× 1062 kb) [file 12248_2020_468_MOESM1_ESM.docx]

## Supplement

**The influence of drug properties and ontogeny of transporters on pediatric renal clearance through glomerular filtration and active secretion – A SIMULATION-BASED STUDY**

S Cristea^1^, EHJ Krekels^1^, A Rostami-Hodjegan^2,3^, K Allegaert^4,5,6^, CAJ Knibbe^1,7^

^1^ Division of Systems Biomedicine and Pharmacology, Leiden Academic Center for Drug Research, Leiden University, Leiden, The Netherlands

^2^ Simcyp Limited, Sheffield, United Kingdom

^3^ Centre for Applied Pharmacokinetic Research (CAPKR), University of Manchester, Manchester, United Kingdom

^4^ Clinical Pharmacy, Erasmus Medical Center, Rotterdam, Netherlands

^5^ Department of Development and Regeneration, KU Leuven, Leuven, Belgium

^6^ Department of Pharmaceutical and Pharmacological Sciences, KU Leuven, Leuven, Belgium

^7^ Department of Clinical Pharmacy, St. Antonius Hospital, Nieuwegein, The Netherlands

**S1: Retrograde calculation of transporter-mediated intrinsic clearance from adult renal clearance values**

Following an extensive literature search, Scotcher *et al.*[1] published data on renal clearance (CL_R_) of 157 drugs in adults,. These drugs were classified according to the publication of Varma *et al*[2]*.* into (i) compounds with net renal reabsorption (CL_R_ < 0.8 x f_u_ x GFR), (ii) compounds with net renal secretion (CL_R_ > 1.2 x f_u_ x GFR) and (iii) compounds with no net reabsorption or secretion (0.8 x f_u_ x GFR < CL_R_ < 1.2 x f_u_ x GFR). Only findings on the 53 net secretion drugs were used in this analysis[2].

By solving equation [S1] for CL_int,sec_ we obtain [S1A], where all terms are known and all parameter values take adult values.

$CL_{R}=f_{u}\times GFR+\frac{\left( Q_{R}-GFR \right)\times f_{u}\times CL_{int,sec}}{Q_{R}+f_{u}\times\frac{CL_{int,sec}}{BP}}$ [S1]

$CL_{int,sec}=\frac{\left( CL_{R}-f_{u}\times GFR \right)\times Q_{R}}{({(Q}_{R}-GFR)\times f_{u} -(CL_{R}-f_{u}\times GFR)\times\frac{fu}{BP})}$ [S1A]

To get CL_int,T_ we solved equation [S2] for CL_int,T_ and obtained the form in [S2A], where all parameter values take adult values and CL_int,sec_ from equation [S1A] is used in equation [S7A].

$CL_{int,sec}=ont_{T}\times CL_{int,T}\times PTCPGK\times KW$ [S2]

$CL_{int,T}=\frac{CL_{int,sec}}{ont_{T}\times PTCPGK\times KW}$ [S2A]

The CL_int,T_ values obtained for 53 drugs classified as *net secretion* drugs following the retrograde calculation are shown in Figure S1.


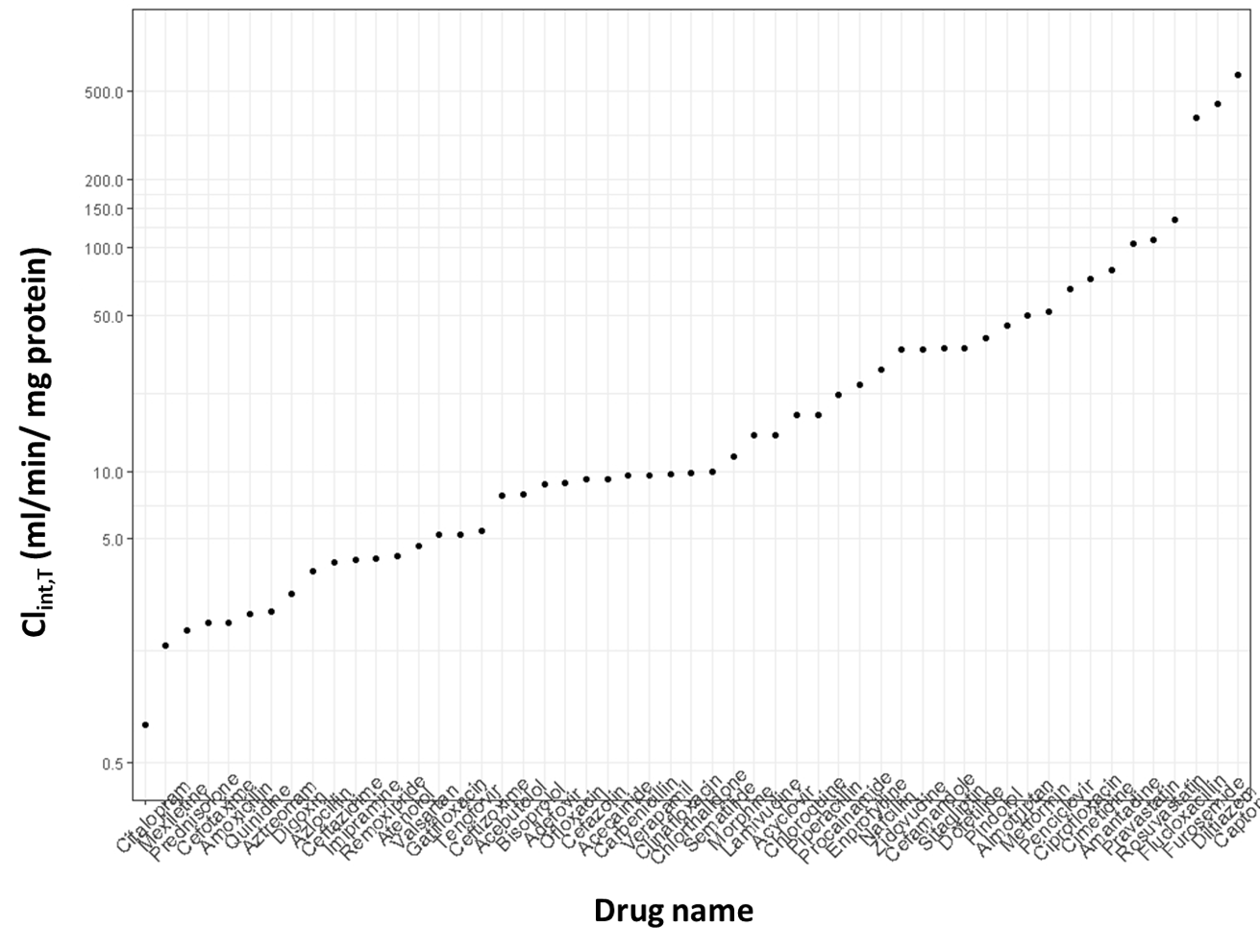


Figure S1 – Intrinsic clearance (CL_int,T_) values obtained for 53 drugs classified as *net secretion* drugs collected from literature. Drugs are ordered by CL_int,T_ values. Y-axis is logarithmic.


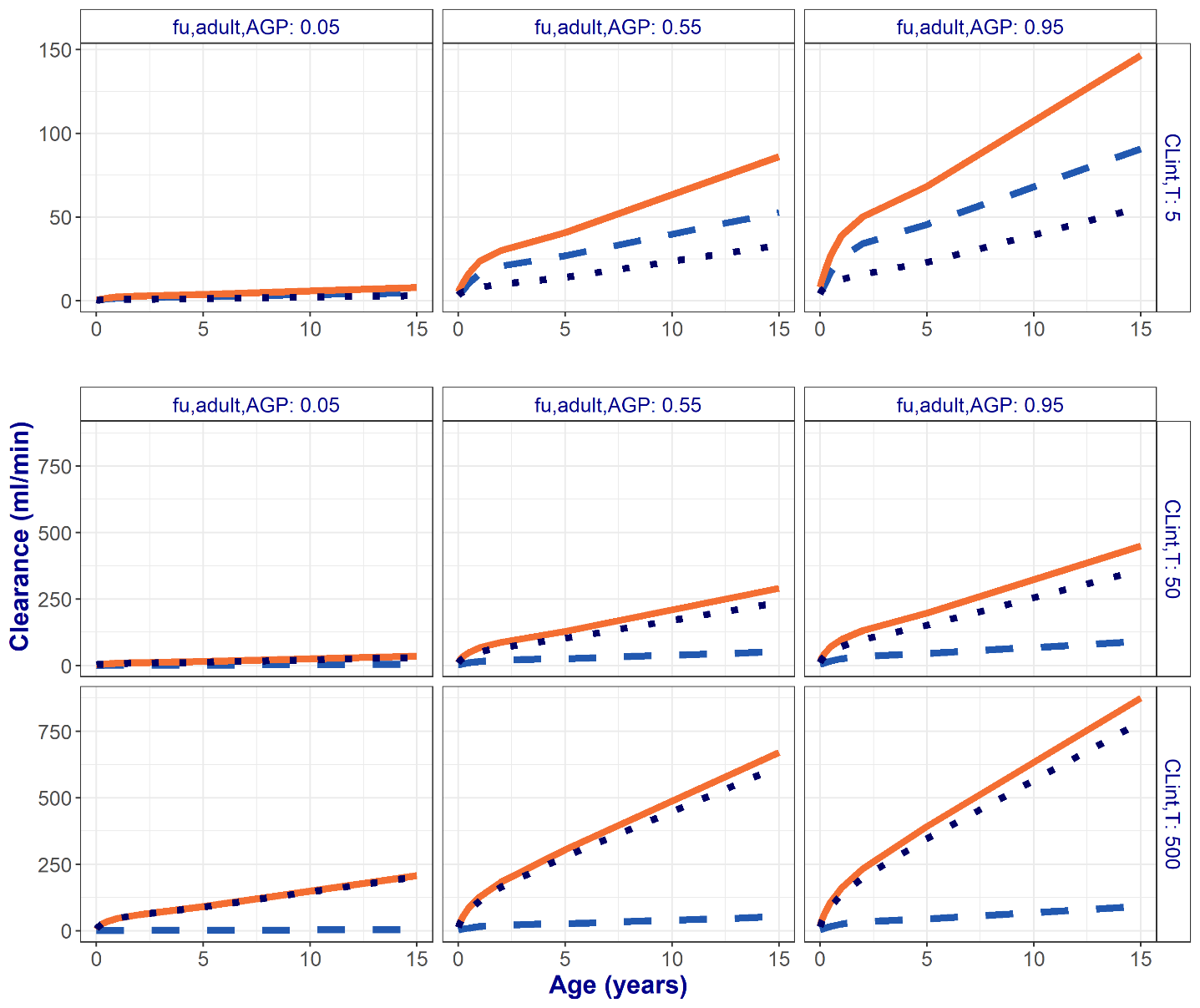


Figure S2 – Developmental changes in total renal clearance (CL_R_ – solid orange lines) and the contribution of glomerular filtration (GF – light blue dashed lines) and active tubular secretion (dark blue dotted lines) vs. age for 9 representative hypothetical drugs. These drugs bind to α-acid glycoprotein (AGP) and have low, medium or high unbound fractions in adults (f_u,adult_ - horizontal panels) that change with age, dependent on the AGP plasma concentrations. Transporter-mediated intrinsic clearance values (CL_int,T_) were assumed to remain constant with age at the indicated values (vertical panels)..Note the different scales on the y-axes for the graphs in the top row (range 0-150 ml/min) compared to middle and bottom row (range 0-750 ml/min).

.


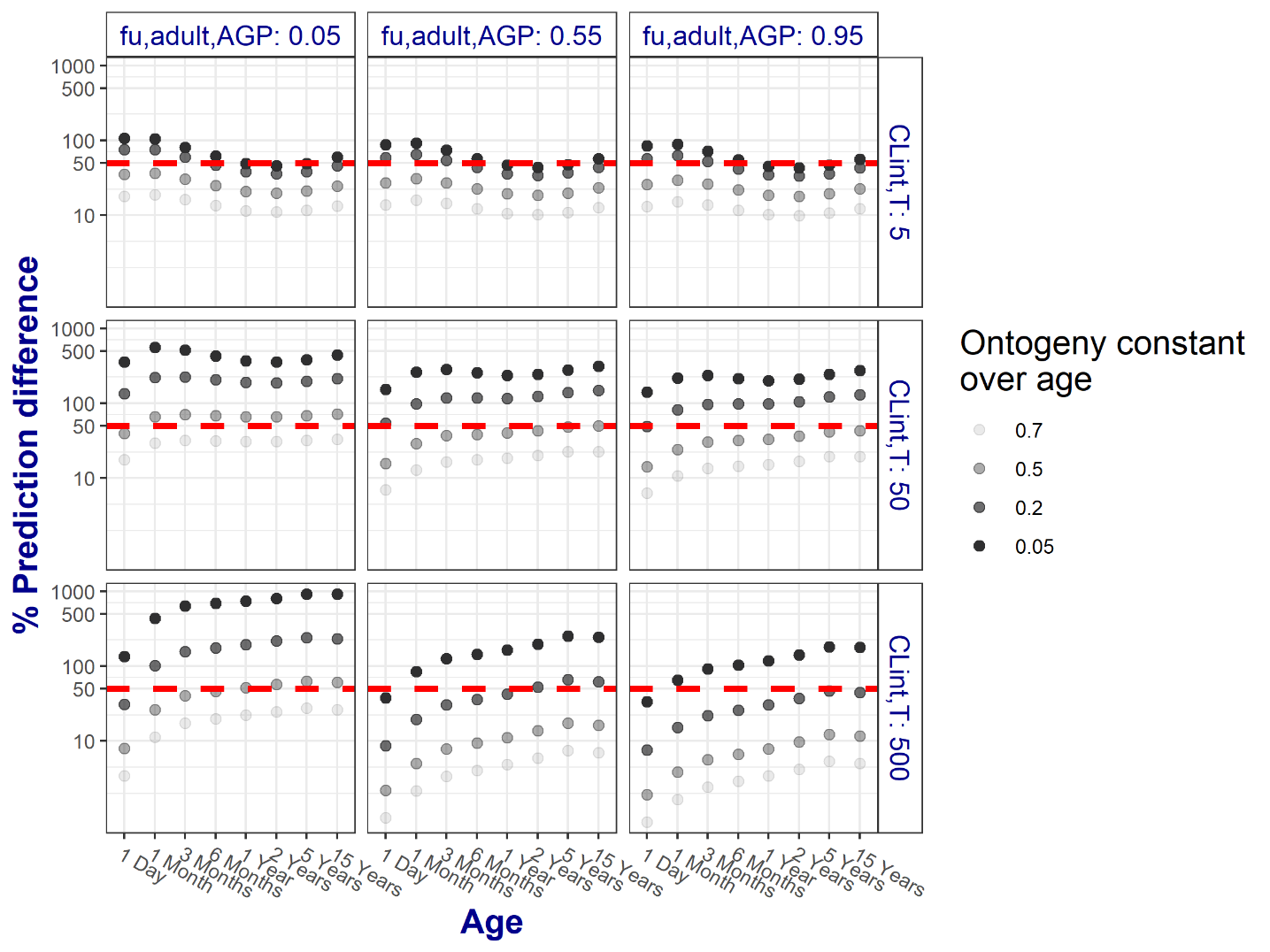


Figure S3 – Percentage Prediction difference (%PD) for 9 representative hypothetical drugs calculated between renal clearance (CL_R_) predictions obtained with the renal PBPK model that included or excluded hypothetical transporter ontogeny (ont_T_) values that remained constant over age. These drugs bind to α-acid glycoprotein (AGP) and have low, medium or high unbound fractions in adults (f_u,adult_ - horizontal panels) that change with age, dependent on the AGP plasma concentrations. Transporter-mediated intrinsic clearance values (CL_int,T_) were assumed to remain constant with age at the indicated values (vertical panels). The colors of the %PD increases with decreasing transporter ontogeny values (ont_T_). The dashed red line represents the threshold of reasonably acceptable CL_R_ prediction of 50%. Results are displayed on a log-log scale.

**References**

1. Scotcher D, Jones C, Rostami-Hodjegan A, Galetin A (2016) Novel minimal physiologically-based model for the prediction of passive tubular reabsorption and renal excretion clearance. Eur J Pharm Sci. https://doi.org/10.1016/j.ejps.2016.03.018

2. Varma MVS, Feng B, Obach RS, Troutman MD, Chupka J, Miller HR, El-Kattan A (2009) Physicochemical determinants of human renal clearance. J Med Chem 52:4844–4852 . https://doi.org/10.1021/jm900403j
